# Development and implementation of a patient assistance fund: a descriptive study

**DOI:** 10.1186/s12913-020-06000-z

**Published:** 2021-01-06

**Authors:** Burke W. Soffe, Justine E. Miranda, Jenny Fang, Daniel G. Epperson, Roberto A. Lara, Hazel L. Williamson, Martin S. Lipsky

**Affiliations:** College of Dental Medicine, Roseman University of Health Sciences, 10895 S. River Front Pkwy, South Jordan, UT 84095 USA

**Keywords:** Health professions education, Access to care, Dental care, Oral health, Patient assistance fund, Patient outcomes assessment, Financial barriers

## Abstract

**Background:**

The purpose of this descriptive study is to outline the Roseman University of Health Sciences (RUHS) College of Dental Medicines’ Patient Assistance Fund development, organization and outcomes. The description and reported results provide insight to others considering similar health professions programs.

**Methods:**

The Patient Assistance Fund (PAF) affords dental students an opportunity to petition for and obtain financial assistance for their most disadvantaged patients. In this study, two sources of data were collected and used with a quantitative analysis for data collected as part of the PAFs operation and a qualitative analysis to evaluate the patient experiences.

**Results:**

A total of 16 student advocates, consisting of 6 males and 10 females from the D3 and D4 classes made 26 presentations to the PAF board committee. The combined amount requested from the PAF was $47,428.00 (“Cost of Treatment Plan”) representing an average request per patient of $1824.15 (range $324.00 to $4070.00). The approved procedures and treatment plans totaled $21,278.36 (“Cost of Approved Procedures”) with an average of $818.40 (range $204.00 to $2434.00) per patient. Patients and students expressed a high degree of satisfaction with the program.

**Conclusions:**

This study provides an overview of the structure, funding sources, expenditures and patient services supported by a dental student managed patient assistance fund. The experiences at RUHS College of Dental Medicine (CODM) suggest that other healthcare professions schools can develop similar type programs that yield benefit both to students and to patients in need.

**Supplementary Information:**

The online version contains supplementary material available at 10.1186/s12913-020-06000-z.

## Background

The significant improvement in the oral health of Americans over the past 50 years is a public health success story [1]. However, striking disparities in dental disease and access to care still exist in the United States. Among the barriers to care, financial limitations remain the most significant obstacle [2–4]. Global statistics link access to regular dental care directly to annual income [5]. In the US, a 2020 Surgeon General’s report noted that socioeconomic factors are significant risk factors for poor oral health and poverty increases the likelihood of tooth loss. In addition, individuals living in poverty account for the highest percentage of people who did not get dental care due to cost [6, 7]. According to Vujicic et al., 59% of adults who had not visited the dentist within the past 12 months cite cost as the reason, more than any other type of health care [4]. Risks of delaying oral health care can lead to needless pain, increased time off from school or work, advanced dental disease including untreated caries, periodontal disease, and tooth loss [8]. Evidence suggests that marginalized patients often seek treatment in emergency departments for problems that might have been prevented given access to adequate oral health care [9].

Dental schools, as safety net facilities, offer a more affordable option for dental care when compared to private practice dental offices [10]. As a result, it is not surprising that the economically disadvantaged are the most frequently reported special population receiving care at dental school clinics in the United States [11]. However, even with reduced costs, some patients still cannot afford even the most simplified and less costly treatment plans [12].

In 2017, a group of students at Roseman University of Health Sciences (RUHS) College of Dental Medicine (CODM) in South Jordan, Utah wanted to improve access at the CODM student clinic by reducing financial barriers to care. To achieve this goal, the students searched for ideas from models used at other institutions such as the dental student founded and managed club at Oregon Health Sciences University designed in 2013 “to change the way underserved populations interact with the world by empowering patients to manage their own health through direct dental health promotion” [13]. RUHS students then approached the CODM and University leadership with the concept of creating their own student managed Patient Assistance Fund (PAF) to provide financial assistance to the clinic’s most financially vulnerable patients. Proposed program goals were 4-fold:

- Provide assistance to dental patients “of record” at Roseman University College of Dental Medicine (CODM) clinic
- Alleviate financial barriers and improve access to dental care
- Cultivate a humanistic and person-centered care approach by allowing student dentists to identify and advocate for patients in financial need
- Enhance student education and clinical experience

By lowering the financial burden of dental care, the student managed PAF helps indigent patients achieve a level of oral health that would otherwise not be unattainable. Under the guidance of a faculty mentor, student dentists advocate to a group of their peers for patients who would otherwise refuse treatment due to cost. This advocacy role fosters an open dialogue between student dentists and patients regarding treatment needs, oral health, and access to care. Peer group decisions regarding financial awards also give students an appreciation and skills regarding maximizing limited resources.

The purpose of this descriptive study is to outline the RUHS CODM PAF’s development, organization and outcomes. To our knowledge, this represents the first published comprehensive description and associated outcomes of a dental student-driven patient assistance fund. The description and reported results should provide insight to others considering similar programs.

## Methods

### IRB statement

The study protocol was reviewed and approved by the Roseman University of Health Sciences Institutional Review Board with an assigned study number of 1,513,182.

### Setting

Roseman University of Health Sciences (RUHS) is a private, nonprofit health sciences university with campuses in Nevada and Utah. RUHS offers degrees in nursing (BSN), dentistry, pharmacy, and a master’s in business administration as a dual degree with pharmacy and dentistry. All programs are fully accredited and employ a mastery-based model, using criteria and curriculum relevant to their discipline. RUHS College of Dental Medicine’s (CODM) undergraduate dental program is located in South Jordan, Utah, part of the Salt Lake City metropolitan area. The CODM is a 4-year program and enrolled its inaugural class of 64 in 2011. Currently RUHS admits 100 new students per year and with a total enrollment of 366 students.

As part of the CODM operations, the dental student clinic offers comprehensive dental care to the local community. Students work in teams under the supervision of dental faculty with one faculty supervisor for approximately six chairs. In the year 2019, the patient care clinics had approximately 36,000 patient visits and while the student clinic treats a broad spectrum of individuals, it predominantly serves an economically disadvantaged population. Sixty-nine percent of the patient pool falls within the age range of 18–64 years old with 16% under 17 years of age and 15% over age 65. Approximately half are Hispanic and 59% of the active are uninsured, 8% have private insurance, and 3% have Medicaid. Twenty-six percent of the active patient pool currently take advantage of the Roseman Membership Plan which is a program offered to patients without dental insurance to provide discounts on regular dental fees. To increase access to those in need, the clinic offers pro bono services to patients referred by several non-profit community groups. For self-pay patients, the clinic offers services at approximately half the cost of private offices.

### Patient assistance fund

Despite lower costs and pro bono services for community members without resources, several students recognized that many patients still faced financial barriers to treatment plans. To address this gap in services, students in consultation with the CODM leadership conceived and developed the Patient Assistance Fund (PAF). Operationalized in August 2018, the PAF affords dental students an opportunity to petition for and obtain financial assistance for their most disadvantaged patients. Under its bylaws (available on request - bsoffe@roseman.edu), the PAF established award guidelines and a process to elect officers and to form a nine-member governing board consisting of at least one faculty member and at least one representative from each dental class year. Students in good academic standing interested in becoming board members complete an application and are interviewed by the current board. By placing students at the forefront, the PAF process sought to cultivate an appreciation for managing limited resources and to nurture a commitment for caring for the underserved.

In order for a patient to be financially eligible for PAF assistance, the PAF bylaws outlined that an established RUHS patient must meet one of the following criteria:

- Enrollment in a government assistance plan
- Residence in low income housing
- Currently homeless
- Annual income at or below the Utah state poverty levels

### Services

Given its limited resources, the PAF board caps individual awards at $1200 and seeks to direct awards to the clinic’s most financially vulnerable patients. In addition, the PAF gives priority to the following treatments:

- Treatments to establish disease control (caries, periodontal disease, pulpal pathology).
- Treatments to improve dental function by 50% (as calculated by first molar function).
- Treatments to improve oral health.
- Treatment to improve dental aesthetics when it will significantly improve the patient’s quality of life.

### Funding sources

Philanthropic contributions provide the majority of funds for the PAF with a goal of raising and distributing between $10,000 to $15,000 annually. Initial funding came from a $5000 grant from Ultradent Products, Inc. and matching RUHS funds. For the following year (2019), the CODM PAF raised $6451 during its annual thanksgiving week, a week dedicated to soliciting support for RUHS priorities. The PAF also received additional funding from CODM leadership and faculty through matching gifts, payroll deductions and donations made throughout the year. The 2019 class also directed a class gift to the fund.

### Distribution of funds

Accessing PAF support requires that a CODM student identify patients who they believe without financial assistance cannot otherwise afford dental treatment. Patients interested in obtaining financial assistance from the PAF must sign a consent form and complete a self-attestation form documenting their financial need in axiUm, the CODM electronic health record. Students then complete a PAF checklist form describing an approved treatment plan and a short statement advocating for their patient (see Table 1). The checklist helps ensure that patients meet financial criteria and that the committee has the relevant information prior to presentation. Before soliciting the PAF, the committee asks that patients and students exhaust other possible financial assistance options.

**Table 1:** PAF Checklist located in electronic health record (EHR)

Patient Assistance Fund (PAF) Checklist		
Tab 1 – General Information		
Student Name		
Patient Name		
Proposed Treatment Plan/Treatment Codes		
Treatment Plan approved by faculty	Yes	No
Treatment Plan approved by patient	Yes	No
General Consent, Screening, and Comprehensive Exam Consent, PAF Consent all signed	Yes	No
Income Eligibility Requirements Passed	Yes	No
Tab 2 – Patient Circumstances		
Brief synopsis of patient circumstances/why patient should be considered for funding		
Tab 3– Approval or Denial		
Approved	Yes	No
Denial Reasoning		
Request for more information		

**Fig. 1:**
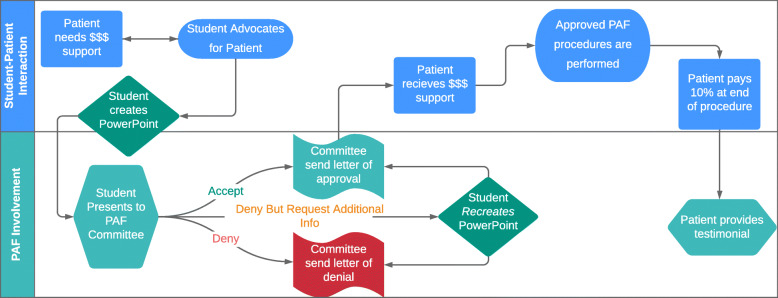
Summary of the PAF Process

After completing the PAF checklist requirements, the student schedules a time to present the patient to the PAF student board. Presentations typically last 15 to 20 min and include the patient’s history, intra- and extra-oral pictures, radiographs and a proposed treatment plan required to control oral disease. Social and financial barriers are also part of the presentation. The board provides a PowerPoint template to guide student presentations and to outline the information required. The presentation guide can be found in Additional file 1. After presenting, the student advocate leaves and the board, with input from their faculty advisor, deliberates about whether to award funds to support treatment. Decisions incorporate both clinical and social factors and can be up to 90% of treatment costs with a $1200 maximum award. On occasion, the board requests additional information before making a decision. Approved patients receive a credit to their account. Further description of the PAF can be found at (link to PAF flyer: https://drive.google.com/open?id=1mkvg3MUpymdTfwfu_LWvNz2UEplW-ANy). Figure 1 summarizes the PAF process.

### Data collection

A PAF student board member enters presentation data including demographics, proposed treatment, award decision and survey responses into an Excel spreadsheet. All participant and student identifiers were removed and the data re-coded to protect participant identity. The key and the de-identified data collection sheet were secured in the researchers’ shared Google Drive with only the faculty advisor able to grant access.

To track PAF awards, the business office created a notation in the patient’s billing record allowing funds to be transferred from the PAF into the patient’s account. The Clinical Business Service Coordinator and PAF treasurer coordinate fund expenditures to allow the PAF board to track expenditures.

A convenience sample of patients who voluntarily agreed to complete a post-treatment survey was used to assess the program. Surveys can be found in Additional file 1. The survey was distributed and collected through Google Forms. The survey sought to evaluate the impact of the PAF supported treatment and consisted of five open ended questions and three Likert style questions asking patients to rank aspects of their experience using a 1 to 10 scale. (survey available on request bsoffe@roseman.edu).

To date, 26 student presentations have been delivered and five post procedural surveys completed by patients.

### Data analysis

In this study, two sources of data were collected and used. Over the period from November 2018 to March 2020, quantitative patient and treatment data collected as part of the PAFs operation and management was uploaded to an Excel file. The Excel Statistical Analysis ToolPak was used to calculate descriptive statistics and to create graphs and tables describing patient characteristics and procedures. Costs were also captured and uploaded to an Excel file and similarly analyzed.

Two authors (BWS and MSL) independently reviewed de-identified qualitative data collected from patients. Using a framework of anticipated themes, each reviewer organized and coded the responses. Then the two reviewers compared their thematic analyses. Differences in analyses were discussed to reach resolution about how they should be categorized. Unanticipated thematic responses were classified as “other” unless more than one student response were similar. In those instances, a new thematic category was added.

## Results

A total of 27 student advocates, consisting of 10 males and 17 females from the D3 and D4 classes made 26 presentations to the PAF board committee. Two examples of cases presented by students, including the outcomes of treatment, can be found in Additional file 2. Twenty different patients were presented during these 26 presentations and 8 presentations were presented by multiple students with 8 re-presentations of the same patient. Six students were involved in re-presenting patients for additional assistance for treatment coverage or a change in treatment. The combined amount requested from the PAF was $47,428.00 (“Cost of Treatment Plan”); representing an average request per patient of $1824.15 (range $324.00 to $4070.00). Since the PAF bylaws allow awards up to 90% of the treatment plan, the maximum financial burden requested from the PAF (“Amount Requested”) was $44,685, an average of $1641.74 (range $291.60 to $3663.00) per patient (see Table 2).

**Table 2:** Financial Data Collected from Nov 2018 - March 2020

Financial Data Collected from Nov 2018 – March 2020		
	Total	Average
Cost of Treatment Plan	$47,428.00	$1824.15 (ranging from $324.00 to $4070.00)
Amount Requested (90% of treatment plan)	$42,685.20	$1641.74 (ranging from $291.60 to $3663.00)
Cost of Approved Procedures	$21,278.36	$818.40 (ranging from $204.00 to $2434.00)
PAF Contribution (Amount Awarded)	$19,150.52	$736.56 (ranging from $183.60 to $2190.60)

The approved procedures and treatment plans totaled to $21,278.36 (“Cost of Approved Procedures”) with an average of $818.40 (range $204.00 to $2434.00) per patient. The amount credited to the patients’ accounts which was up to 90% of the “cost of approved procedures” was $19,150.52, with an average award of $736.56 (range $183.60 to $2190.60) per patient. The board implemented a $1200 maximum shortly after starting the program and therefore a few patients received PAF financial aid in excess of $1200 (see Table 2).

Among the three award designations: full award (PAF covers 90% of the complete treatment plan presented up to $1200), partial award (PAF covers 90% of approved parts of the proposed treatment plan), and no award; the board approved 10 (38.5%) full awards, 10 (38.5%) partial awards, and denied 6 (23.1%) requests. Reasons for not approving funding included unsigned consent forms, inadequate or missing information to assess the treatment plan (e.g. radiographs, periodontal charting, treatment plan, intraoral photographs, etc.) or the patients failing to meet PAF eligibility criteria. The committee also denied some or all of a proposed treatment plan if they felt treatment did not meet the PAF’s criteria of eliminating disease and restoring first molar occlusion function. If no funding was awarded, the PAF board encouraged student dentists to present their patient for reconsideration if circumstances changed. While each of the 26 presentations were treated as distinct data points, eight of them were re-presentations.

**Fig. 2:**
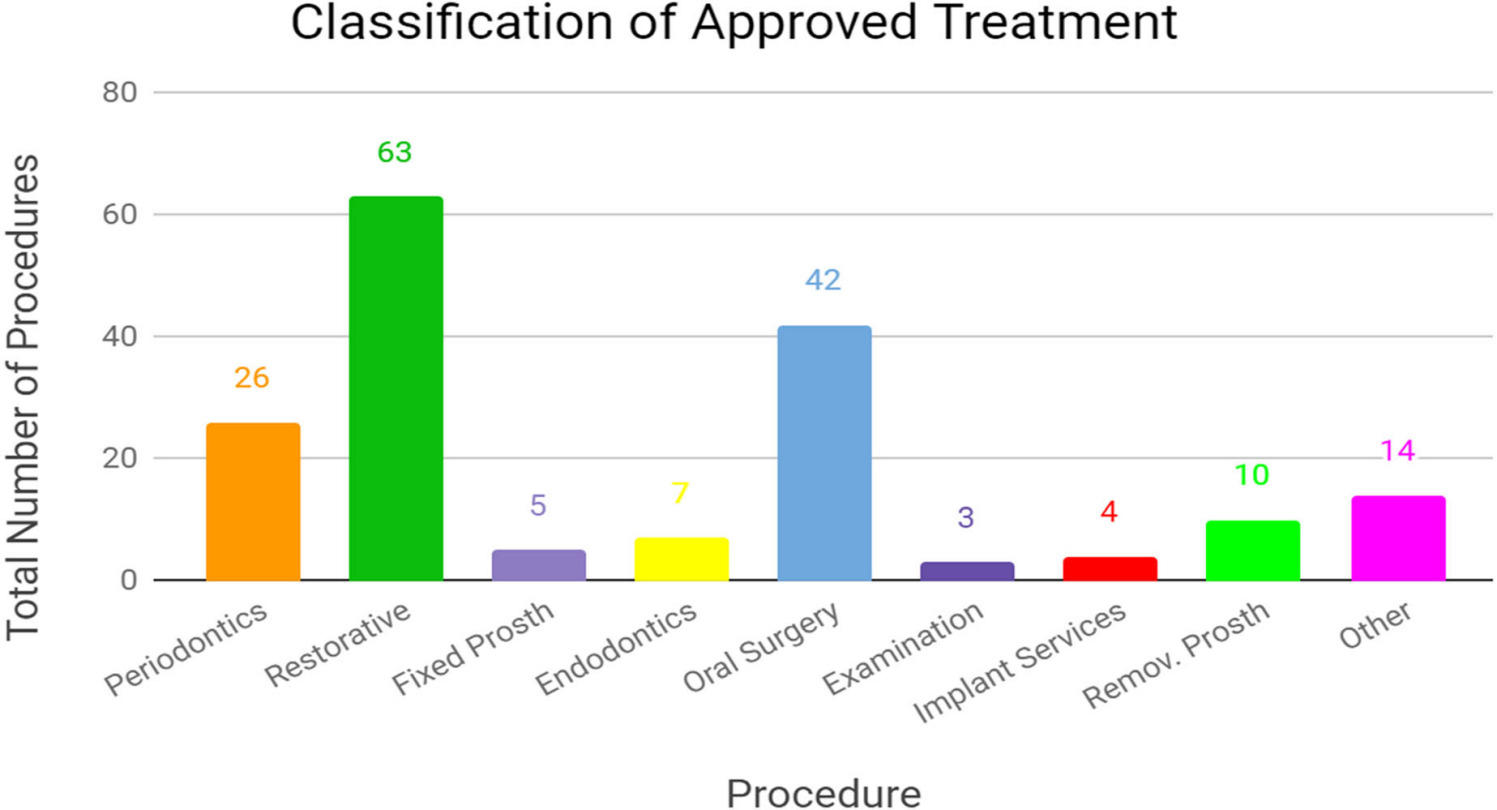
Classification of Approved Treatment

The 20 full awarded and partially awarded treatment plans represented a total of 174 CDT codes. Of the 174 CDT codes, the top three treatment types: restorative treatment (63 codes, 36%), oral surgery (42 codes, 24%), and periodontics (26 codes, 15%) accounted for 75% of the approved procedures (see Fig. 2). Figure 2 lists the eight most commonly used CDT codes.

Only five patients volunteered to complete a post-operative survey. Two patients were Caucasian, two Syrian, and one Hispanic. Three patients were female and two were male. All ranked “receiving financial assistance made it easier to receive dental care” as a “10” on a 1 to 10 Likert scale. When asked to assess their quality of life before dental treatment, the average response was 2 which improved to 8.25 after receiving PAF funded dental treatment. Four patients indicated that finances were the largest barrier to dental care and that they had to refuse the optimal treatment plan or delay treatment due to finances. Table 3 summarizes the patient responses to the open-ended question.

**Table 3:** Patient Interview Responses, *N* = 5

Patient Interview Responses, ***N*** = 5		
**Question**	**Theme**	**# Comments**
What is your largest barrier to receiving dental treatment?	Finances	5
	Language	2
How has receiving PAF financial assistance impacted your quality of life and/or overall health?	Improved smile/ confidence	5
	Ability to eat	3
	Affordability of dental care	3
How would your life be different if you did not receive funding?	Not be able to afford any treatment	2
	Extraction of remaining teeth	1
How has this impacted your relationship to dentistry?	Stronger desire to visit dentist	2

## Discussion

Dental care is far more than just healthy teeth; poor oral health adversely affects nutrition, socialization, communication and overall systemic health [9]. It requires personalized care which addresses the biologic aspects of care as well as psychosocial and socioeconomic factors. Recognizing the importance of financial barriers to restoring oral health, RUHS dental students developed a PAF to support those patients exhibiting both a financial and clinical need. The findings reported here suggest that with the support of a school’s administration, motivated dental students can create and organize a process to successfully manage a charitable fund that supports patient care.

When the PAF began, the board purposely chose a soft launch so that it could adjust policies and processes to unanticipated problems. Early on, the board recognized the need to develop a rigorous process to confirm that only patients demonstrating financial and clinical need would receive financial help. Even with the intentional decision to go slow, it was still surprising that to date only 16 students made presentations. To increase the number of participating students, the board made presentations at both class and faculty meetings and anticipates great participation in the next academic year. Also unexpected was that almost 80% of presentations received an award. One explanation for the high success rate was that the PAF checklist and presentation template helped advocates to identify patients that met financial and treatment criteria and to make compelling presentations.

Over a two-year period, 20 patients received care with an average cost of treatment of $891.60 per patient. In contrast, the ADA in 2013 reported an average per patient expenditure for general dentists of $514 and an average expenditure of $1625 per patient for patients at the 90th percentile of cost [4]. While patients with high dental expenditures represent a relatively small percentage of patients, they arguably represent those with the greatest clinical need. The PAF’s average award of $891.60 against charges at about 50% of the prevailing community dental charges suggests that the PAF met its goal to target those with great clinical need. The costs reported here should also help other institutions estimate direct expenditures for a PAF. While indirect costs are not reported and were absorbed by RUHS, they include items such as faculty supervision, meeting rooms, fund raising, business office accounting costs and IT support.

While helping 20 patients may seem of little consequence, the impact on assisting these patients is highlighted by their survey responses. All surveyed patients stated that finances were the most significant factor influencing their ability to receive dental treatment and expressed their inability to obtain dental care compromised their quality of life. All reported marked improvement after treatment and reported a greater likelihood to seek dental care in the future. Programs that address a high risk person’s oral health needs can limit the progression of oral disease, favorably impact overall health and reduce overall healthcare costs [14–17]. Similarly, evidence suggests additional cost benefit by helping to avoid costly emergency department visits for problems that might either be prevented or cared for in a dental office [9].

Finding the resources to develop and sustain a PAF is likely to be a challenge for institutions considering a similar program. The RUHS CODM PAF relies on philanthropic donations and benefitted from institutional support including assistance from the University’s development office. While fundraising to date has been successful, the future challenge will be to sustain and ideally increase total annual awards. To assure success, the PAF team works closely with the development office to develop strategies to solicit additional corporate and external funding sources. Other institutions considering a PAF will benefit from developing strategies for sustainability and should consider engaging their development team even during planning phases.

### Limitations

There are several limitations to this descriptive research study. First, the sample size is small and represents a single dental school. However, in a future project, the PAF board plans to increase awareness of the PAF to students with the goal of increasing student participation. Furthermore, the PAF board plans to systematically study the programs impact on the student body and student advocates. Second, while all patients surveyed reported high satisfaction with the program the number of respondents was small. However, the result is consistent with the literature reporting correspondingly high levels of satisfaction expressed by patients receiving free care in a student clinic [18]. We also plan a future study to examine patient satisfaction in greater depth.

## Conclusion

This study provides an overview of the structure, funding sources, expenditures, student and patient outcome, and patient services supported by dental student managed patient assistance fund. The experiences at RUHS CODM suggest that other dental schools can develop similar type programs that yield benefit both to students and to patients in need.

## Supplementary Information

**Additional file 1.**

**Additional file 2.**

## Data Availability

The datasets used and/or analyzed during the current study are available from the corresponding author on reasonable request.

## Abbreviations

RUHS: Roseman University of Health Sciences
CODM: College of Dental Medicine
PAF: Patient Assistance Fund
CDT: Current Dental Terminology. Current Dental Terminology (CDT) is a code set with descriptive terms developed and updated by the American Dental Association (ADA) for reporting dental services and procedures to dental benefits plans
EHR: Electronic Health Record

## References

[1] Department of Health and Human Services, Office of Disease Prevention and Health Promotion (2020). Healthy people.

[2] Bloom B, Simile CM, Adams PF, Cohen RA (2012). Oral health status and access to oral health care for U.S. adults aged 18–64; National Health Interview Survey, 2008. National Center for Health Statistics. Vital Health Stat.

[3] Yarbrough C, Nasseh K, Vujicic M. Why adults forgo dental care: evidence from a new national survey. In: Health policy institute research brief: American Dental Association; 2014. At: http://www.ada.org/~/media/ADA/Science%20and%20Research/HPI/Files/HPIBrief_1114_1.ashx. Accessed: 15 Mar 2020.

[4] Vujicic M, Buchmueller T, Klein R (2016). Dental care presents the highest level of financial barriers, compared to other types of health care services. Health Aff.

[5] Freeman R (1999). Barriers to accessing dental care: patient factors. Br Dent J.

[6] National Institute of Dental and Craniofacial Research (2002). Oral health in America: a report of the surgeon general—executive summary.

[7] 2020 Surgeon General’s Report, Oral Health in America: Advances and Challenges. International Association for Dental Research 97th General Session. At: https://www.nidcr.nih.gov/sites/default/files/2019-08/SurgeonGeneralsReport-2020_IADR_June%202019-508.pdf. Accessed 19 Oct 2020.

[8] Aarabi G, Valdez R, Spinler K, Walther C, Seedorf U, Heydecke G, König HH, Hajek A (2019). Determinants of postponed dental visits due to costs: evidence from the survey of health, ageing, and retirement in Germany. Int J Environ Res Public Health.

[9] Shortridge EF, Moore JR (2010). Use of emergency departments for conditions related to poor oral health care.

[10] Edelstein B (2010). The dental safety net, its workforce, and policy recommendations for its enhancement. J Public Health Dent.

[11] Doris JM, Davis E, Du Pont C, Holdaway B (2009). Social work in dentistry: the CARES model for improving patient retention and access to care. Dent Clin N Am.

[12] Zittel-Palamara K, Fabiano J, Davis E, Waldrop D (2005). Improving patient retention and access to oral health care: the CARES program. J Dent Educ.

[13] Oregon Health Sciences University School of Dentistry. https://www.ohsu.edu/school-of-dentistry/community-dentistry-education. Accessed 20 Oct 2020.

[14] Griffin A (2012). An integrated approach to healthy aging: peer reviewed. Am J Public Health.

[15] Oakes D., Monopoli M., Medicare dental benefit will improve health and reduce health care costs. Health Affairs Blog 2019. DOI:10.1377/hblog20190227.354079. At: https://www.healthaffairs.org/do/10.1377/hblog20190227.354079/full/. Accessed 20 Oct 2020.

[16] Jeffcoat MK, Jeffcoat RL, Gladowski PA, Bramson JB, Blum JJ (2014). Impact of periodontal therapy on general health: evidence from insurance data for five systemic conditions. Am J Prev Med.

[17] Nasseh K, Vujicic M, Glick M (2016). The relationship between periodontal interventions and healthcare costs and utilization. Evidence from an integrated dental, medical, and pharmacy commercial claims database.

[18] Lawrence D, Bryant T, Nobel T, Dolansky M, Singh M (2015). A comparative evaluation of patient satisfaction outcomes in an interprofessional student-run free clinic. J Interprof Care.

