# Supplementary material for: Development and implementation of a patient assistance fund: a descriptive study
Source: BMC Health Serv Res. 2021 Jan 6;21:14. doi: 10.1186/s12913-020-06000-z (PMC7789741; doi:10.1186/s12913-020-06000-z)
Supplement: Supplementary file 1 — Additional file 1. [file 12913_2020_6000_MOESM1_ESM.docx]

Additional file 1 - Presentation Guide and Post-Op Presentation Guide including Survey Questions

Presentation Template

1. Patient Information
   1. Age
   2. Chart number
   3. Patient history
      1. Start date
      2. Number of appointments at Roseman CODM
      3. Is the patient reliable?
   4. Medical history
2. Dental History
   1. Chief concern
   2. Dental history leading up to this moment
3. Approved Phase 2 Treatment
   1. All specialist consults completed
   2. All clinical tests completed
      1. Diagnosis complete
   3. Screenshot of complete perio chart
4. Radiographs (FMX)
5. Intraoral Photos
6. Estimate of costs
   1. Specific details of treatment plan
   2. Picture of treatment plan
   3. Total cost of treatment plan
7. Who is this patient to you?
   1. Describe student’s relationship with patient
   2. Give patient humanistic aspects

Post Op Presentation

1. Slide 1 – Radiographs (FMX)
2. Slide 2 – Pre-op intraoral photos
3. Slide 3 – Post-op intraoral photos
4. Survey questions
5. What is your gender, race, ethnicity?
6. What is your largest barrier to receiving dental treatment?
7. In the past 12 months, have you refused the optimal treatment option or delayed a treatment plan due to cost of dental care?
8. On a scale from 1-10 (1-not at all, 10-a lot), how has receiving financial aid made it easier for you to receive dental care?
9. On a scale from 1-10 (1-pain/low self confidence, 10-pain free/confident/happy), indicate your quality of life before receiving PAF funded dental treatment.
10. On a scale from 1-10 (1-pain/low self confidence, 10-pain free/confident/happy), indicate your quality of life after receiving PAF funded dental treatment.

Video testimonial

- Questions for the patient
  - How has receiving PAF financial assistance impacted your quality life and/or overall health?
  - Would you be able to pursue treatment without assistance from PAF? How would your life be different if you did not receive funding?
  - How has this impacted your relationship with dentistry?
- Questions for the student
  - How has this process increased your social awareness and improved your clinical experience?
