# Supplementary material for: Development and implementation of a patient assistance fund: a descriptive study
Source: BMC Health Serv Res. 2021 Jan 6;21:14. doi: 10.1186/s12913-020-06000-z (PMC7789741; doi:10.1186/s12913-020-06000-z)
Supplement: Supplementary file 2 — Additional file 2. [file 12913_2020_6000_MOESM2_ESM.docx]

Additional file 2 - Sample Patient Cases

Patient Impact #1:

TS, a patient at Roseman CODM in the 45-64-year-old age range, reports that the largest barrier to receiving dental care is finances and states “I live off disability alone and I live with my family.” TS needed a dental cleaning, nine surgical extractions, two quadrants of alveoloplasty (removing bone to prepare the dental arch for a complete denture), and an immediate maxillary denture and was able to complete treatment through the help of the Patient Assistance Fund. After treatment, TS stated, “I came to Roseman because my medications were breaking down my teeth and I was not able to eat foods. This was hindering my health due to issues with my digestive tract. Since getting my new teeth I have been able to eat a lot better.” Additionally, TS stated, “I would not have been able to afford the [dental treatment] without help from the PAF or Medicaid. I was lucky. I would not be able to eat or like my smile without it.” The student advocate for TS reported, “It was wonderful working with the PAF to help this patient in need afford dental treatments and make it to a place where [TS] ‘liked [their] smile again.’ Without resources like the PAF, patients like TS would not be able to get the treatment they need for a good quality of life.”

Patient impact #2:

HD, a patient at Roseman CODM in the 45-64-year-old age range, reported to the CODM with the chief complaint “I would like to get my teeth replaced so I can eat without choking. I can’t chew food properly.” Additionally, HD reported “I have wanted to take care of my teeth for a very long time, but I just cannot afford to pay for dental care as my disability payment goes almost entirely to rent.” HD was in a terrible car accident in 2000 that caused serious injuries including titanium plates and neck screws. This injury left HD unable to work with limited mobility and the need to walk with the assistance of a cane. Prior to treatment, HD needed five surgical extractions of remaining maxillary teeth, four restorations to remove dental caries, a dental cleaning, alveoloplasty on the upper right and upper left, an immediate maxillary denture, and a mandibular partial denture. With the assistance of the Roseman Patient Assistance Fund, HD was able to complete treatment and be restored to oral health. Following treatment, HD referenced the assistance from PAF and stated, “I wouldn’t have been able to get this done without it… I couldn’t afford it… You need teeth to chew and I’ll finally be able to chew food and not choke.”
